# Editorial: Thyroid dysfunction in pregnant or reproductive-age women

**DOI:** 10.3389/fendo.2023.1195950

**Published:** 2023-04-12

**Authors:** Tuija Männistö, Creswell J Eastman

**Affiliations:** ^1^ NordLab, Oulu, Finland; ^2^ Translational Medicine Research Unit, Faculty of Medicine, University of Oulu, Oulu, Finland; ^3^ Westmead Clinical School, Faculty of Medicine and Health, The University of Sydney, Darlington, NSW, Australia

**Keywords:** thyroid, pregnancy, TSH, autoimmune disease, hypothyroidism

Maternal thyroid dysfunction can affect a woman’s ability to conceive, maintain a healthy pregnancy, and even affect her child in the long term. In this topic of Frontiers in Endocrinology, d’Assunção et al. performed a single center retrospective data analysis with a systematic review and meta-analysis on the effect of thyroid function on assisted reproduction outcomes. Their study found, reassuringly, that the women in their area undergoing IVF/ICSI with TSH levels between 0.5 to 4.5 mIU/L had similar outcomes despite their initial TSH concentration. In the systematic review and meta-analysis, including 17 studies, similar rates of clinical pregnancies were observed in women with TSH between 0.5 to 2.49 mIU/L and 2.5 to 4.5 mIU/L. However, the included studies had high heterogeneity and publication bias was additionally indicated. Therefore, further studies are needed to guide the optimal level of TSH concentration among women undergoing assisted reproductive technologies and to guide clinicians on whom to treat. Right now, those treating women undergoing assisted reproduction should adhere to the 2017 American Thyroid Association guidelines for the diagnosis and management of thyroid disease during pregnancy and postpartum (1) and treat women with subclinical hypothyroidism with levothyroxine (LT4) and aim for a TSH goal of <2.5 mIU/l.

The thought-provoking article by Fitzgerald et al. “*The association of new concepts of the assessment of the thyroid state of pregnant women*” challenges orthodox beliefs and clinical diagnostic practices as the authors question the adherence to the measurement of serum TSH concentrations in preference to serum Free T4 concentrations for defining thyroid status in pregnant women. They are critical of thyroid hormone replacement therapy being prescribed for gestational subclinical hypothyroidism based on maternal serum TSH levels, and they argue that reliance on serum Free T4 is a more appropriate diagnostic practice. Most authoritative guidelines developed for the diagnosis and management of thyroid disorders in pregnancy caution against reliance on Free T4 measurements in pregnancy because of the lack of reproducibility and validity of automated immunoassays from which the results are derived. Indeed, skepticism about the entity of “isolated hypothyroxinemia” in pregnancy was sufficient for the American Thyroid Association guidelines to conclude that this entity should not be treated in pregnancy.

Tańska et al. emphasized in their mini-review the role of maternal thyroid autoimmunity on fertility and obstetric outcome. They concluded that data on the effects of thyroid autoimmunity on fertility are divergent, but summarized data seems to point towards decreased fertility among those women with thyroid autoimmunity. The review does summarize that women with thyroid autoimmunity have a higher risk of miscarriage and other adverse obstetric outcomes. They also sum up the current treatment options based on literature, which is scarce, unfortunately.

There are multiple published systematic reviews and meta-analyses of the possible adverse effects of gestational subclinical hypothyroidism (SCH) on pregnancy and the offspring, but few have examined the potential benefits of LT4 therapy on the outcome of pregnancy. Current guidelines are frequently indecisive or conflicting, recommending for or against replacement therapy, leaving clinicians confused about the management of these patients. In this contribution, “*The impact of levothyroxine therapy on pregnancy, neonatal and childhood outcomes of subclinical hypothyroidism during pregnancy: an updated systematic review, meta-analysis, and trial sequential analysis*” Jiao et al. targeted the literature based on the latest diagnostic criteria provided by the American Thyroid Association (ATA) 2017 guidelines. This updated systematic review and meta-analysis also included a trial sequential analysis. The outcome was disappointing in that the results were inconclusive, as they state they found no evidence of the benefit of LT4 therapy on pregnancy and childhood outcomes. However, they did report trends towards decreased adverse outcomes, such as preterm delivery and miscarriage, leaving the door open for further well-conducted, sufficiently powered clinical trials to answer these questions in the future.

Zhang et al. studied if triglyceride concentrations coupled with low free thyroxine concentration increase the risk of gestational diabetes. Both high triglycerides and thyroid dysfunction alone seem to be associated with gestational diabetes (2, 3). The current study showed in a population of over 40,000 women that the association may interact, and those with low free thyroxine and high triglyceride concentrations had up to 2/4 increased risks of gestational diabetes, compared to those with adequate free thyroxine and low triglycerides. This study showed that the interplay between different risk factors is important, and the risks should be considered as a whole.

Furthermore, the study by Li et al. showed in a cohort of nearly 3,000 Chinese mother-newborn pairs how maternal thyroid function is associated with neonatal TSH levels. Although their study focused on neonates during their first week of life, the study adds to the literature showing that maternal thyroid function during pregnancy may have an even long-term effect on the thyroid function of the child (4).

## Author contributions

TM and CE have drafted the editorial and approved the final version for submission.
